# Differentiation of human pluripotent stem cells into two distinct NKX6.1 populations of pancreatic progenitors

**DOI:** 10.1186/s13287-018-0834-0

**Published:** 2018-04-03

**Authors:** Idil I. Aigha, Bushra Memon, Ahmed K. Elsayed, Essam M. Abdelalim

**Affiliations:** 1 Diabetes Research Center, Qatar Biomedical Research Institute, Hamad Bin Khalifa University, Qatar Foundation, Education City, Doha, Qatar; 2 Department of Anatomy and Embryology, Faculty of Veterinary Medicine, Suez Canal University, Ismailia, 41522 Egypt

**Keywords:** hESCs, hiPSCs, Pancreatic development, Transcription factors, Pancreatic progenitors, β cells

## Abstract

**Background:**

The expression of a specific combination of transcription factors (TFs) in the multipotent progenitor cells (MPCs) is critical for determining pancreatic cell fate. NKX6.1 expression in PDX1^+^ MPCs is required for functional β cell generation. We have recently demonstrated the generation of a novel population of human pluripotent stem cell (hPSC)-derived MPCs that exclusively express NKX6.1, independently of PDX1 (PDX1^−^/NKX6.1^+^). Therefore, the aim of this study was to characterize this novel population to elucidate its role in pancreatic development.

**Methods:**

The hPSCs were exposed to two differentiation protocols to generate MPCs that were analyzed using different techniques.

**Results:**

Based on the expression of PDX1 and NKX6.1, we generated three different populations of MPCs, two of them were NKX6.1^+^. One of these NKX6.1 populations coexpressed PDX1 (PDX1^+^/NKX6.1^+^) which is known to mature into functional β cells, and an additional novel population did not express PDX1 (PDX1^−^/NKX6.1^+^) with an undefined role in pancreatic cell fate. This novel population was enriched using our recently established protocol, allowing their reorganization in three-dimensional (3D) structures. Since NKX6.1 induction in MPCs can direct them to endocrine and/or ductal cells in humans, we examined the coexpression of endocrine and ductal markers. We found that the expression of the pancreatic endocrine progenitor markers chromogranin A (CHGA) and neurogenin 3 (NGN3) was not detected in the NKX6.1^+^ 3D structures, while few structures were positive for NKX2.2, another endocrine progenitor marker, thereby shedding light on the origin of this novel population and its role in pancreatic endocrine development. Furthermore, SOX9 was highly expressed in the 3D structures, but cytokeratin 19, a main ductal marker, was not detected in these structures.

**Conclusions:**

These data support the existence of two independent NKX6.1^+^ MPC populations during human pancreatic development and the novel PDX1^−^/NKX6.1^+^ population may be involved in a unique trajectory to generate β cells in humans.

**Electronic supplementary material:**

The online version of this article (10.1186/s13287-018-0834-0) contains supplementary material, which is available to authorized users.

## Background

Diabetes is a metabolic disorder characterized by chronic hyperglycemia due to progressive loss or impaired function of pancreatic β cells. Type 1 diabetes (T1D) is characterized by a β cell loss due to autoimmune inflammatory-mediated β-cell apoptosis with the subsequent loss of insulin secretion. The hyperglycemia in type 2 diabetes (T2D) is a consequence of β-cell failure in the setting of insulin resistance in the peripheral tissues [1, 2]. Pancreatic β-cell transplantation therapy has great potential for treating T1D and advanced cases of T2D. To date, transplantation of whole pancreas or isolated islet cells from cadavers remains the most effective approach for reversing hyperglycemia in diabetic patients. However, this approach has limitations in terms of the necessity of immunosuppressive drugs and the availability of matching donors [3, 4]. Generation of pancreatic β cells from human pluripotent stem cells (hPSCs) has recently attracted much attention. hPSCs, including human embryonic stem cells (hESCs) and human induced PSCs (hiPSCs) can provide an unlimited supply of pancreatic β cells in vitro, which have great potential to be used for transplantation therapy and to generate in vitro models for studying diabetes (see reviews [5, 6]).

During human development, all adult pancreatic cells originate from the same multipotent pancreatic progenitor cells (MPCs) that express a group of transcription factors (TFs), including PDX1, SOX9, FOXA2, NKX6.1, HNF6, and PTF1a [7, 8]. NKX6.1 and PTF1a are expressed specifically in the pancreas, but other TFs (PDX1, SOX9, FOXA2, and HNF6) are not limited to the pancreas as they are also expressed in the duodenum, stomach, and liver [9]. The coexpression of PDX1 and NKX6.1 in the MPC stage determines the maturation and functionality of pancreatic β cells [10–13]. Based on the expression of key TFs, there are two types of MPCs that appear during development: 1) MPCs expressing both TFs, PDX1 and NKX6.1, which can differentiate into monohormonal (glucose-responsive) β cells; and 2) MPCs expressing only PDX1, which can differentiate into polyhormonal (glucose-unresponsive) endocrine cells. Generation of the two different MPC populations in vitro has been demonstrated using hPSCs [10, 11, 14]. It has also been shown that transplantation of MPCs (PDX1^+^/NKX6.1^+^) can reverse diabetes in animal models [11, 12, 15]. hESC-derived PDX1^+^/NKX6.1^+^ progenitors are currently being used in clinical trials for T1D patients [16, 17]. Recently, only a few studies have successfully demonstrated the ability of hPSCs to differentiate into functional pancreatic β cells in vitro [13, 18, 19]. However, differentiation efficiency of the in vitro protocols and maturity of the generated β cells remain the main problems facing this field (see reviews [6, 20]). Therefore, extensive studies are required to further dissect signaling pathways and transcriptional regulatory networks governing human pancreas development to be able to establish a reproducible differentiation system for generation of functional β cells in vitro [20].

There is accumulating evidence indicating that NKX6.1 is necessary during pancreas development and later becomes required and restricted to β cells [20–22]. NKX6.1 expression has not been detected in glucagon-secreting α cells of the human adult pancreas [22] as well as those generated from the hPSC-derived PDX1^+^ MPCs [14]. Previous studies have consistently reported that NKX6.1 expression in hPSC-derived MPCs is exclusively colocalized with PDX1 expression [10, 13, 15, 18, 19], suggesting the existence of only one type of NKX6.1^+^ MPC population. However, we recently reported the in vitro generation of a novel population of MPCs that express NKX6.1 in the absence of PDX1. Herein, we characterize this novel population suggesting that at least some of the unique PDX1^−^/NKX6.1^+^ MPC population may be precursors for endocrine pancreatic cells. The data presented here set the bar for defining new roles for NKX6.1, which is one of the key TFs involved in human pancreatic β-cell development.

## Methods

### Culture of human pluripotent stem cells

hESC (H1) and hiPSC (IMR90) lines were obtained from WiCell Research Institute (Maddison, USA). Both cell lines were cultured and maintained in 5% CO_2_ at 37 °C in chemically defined mTesR1 medium (Stem Cell Technologies, Canada) on cell culture plates previously coated with 1:80 dilution of Matrigel (Corning, USA). Cells were passaged when the confluence reached about 60–70% as the cells were dissociated by incubation with ReLeSR (Stem Cell Technologies, Canada) for 2–4 min or until detachment of the colony borders, and then the cells were collected and resuspended in mTesR1 medium supplemented with 10 μM Y-27632 (Rock inhibitor) (Stemgent, USA) for first 24 h of passaging.

### Differentiation of human pluripotent stem cells into pancreatic progenitors

**Fig. 1:**
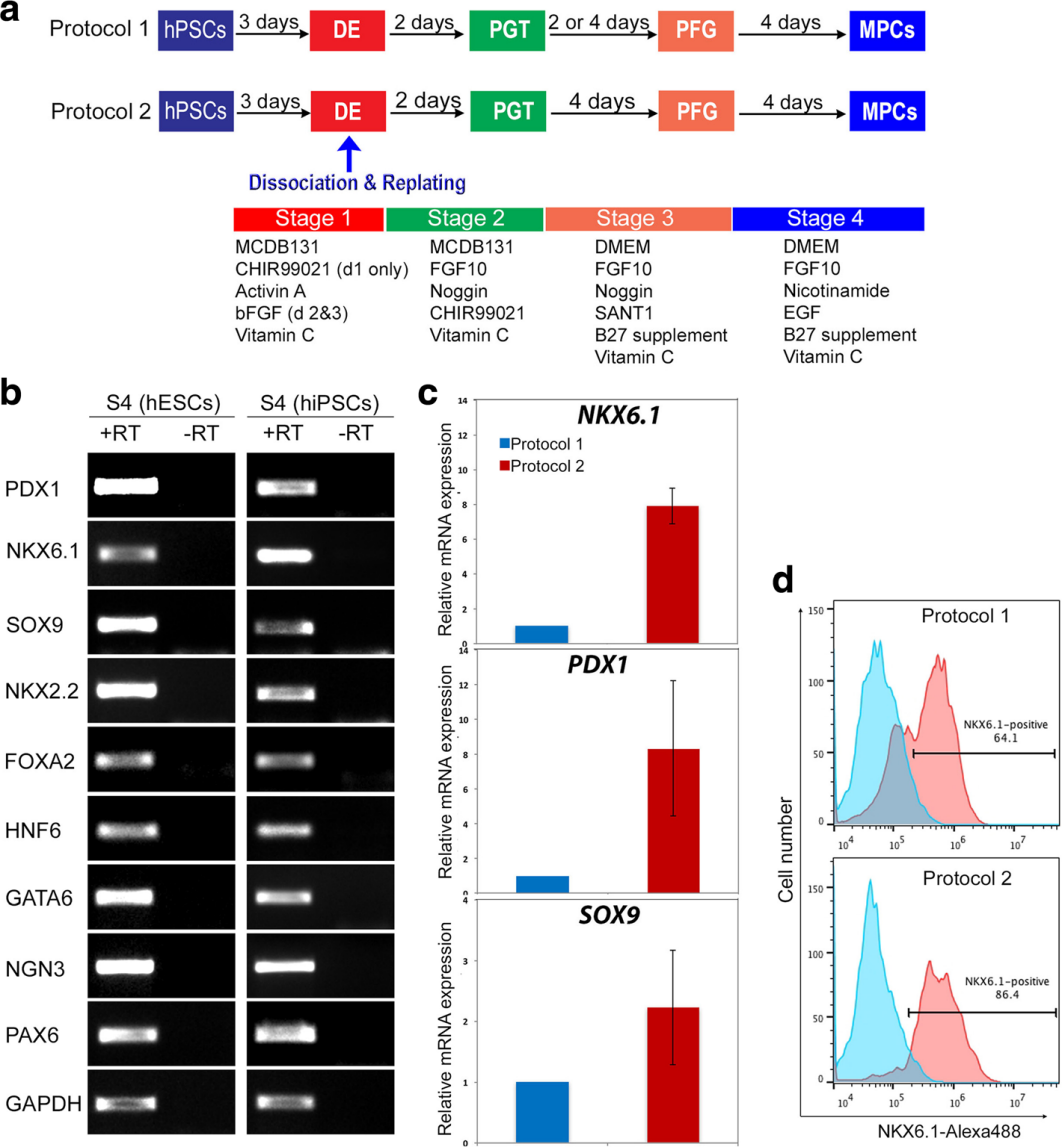
Generation of human pluripotent stem cell (hPSC)-derived multipotent pancreatic progenitors (MPCs). **a** Schematic summary of the protocols used to generate MPCs from hPSCs. hPSCs were differentiated through four stages, including definitive endoderm (DE), primitive gut tube (PGT), pancreatic foregut (PFG), and MPCs. **b** RT-PCR analysis of MPCs derived from human embryonic stem cells (hESCs) and induced hPSCs (hiPSCs) at the end of stage 4 (S4) using protocol 1 showing the expression of the MPC and early endocrine progenitor markers (*PDX1*, *NKX6.1*, *SOX9*, *NKX2.2*, *GATA6*, *PAX6*, *NGN3*, and *HNF6*). +RT indicates samples with reverse transcriptase, and –RT indicates samples without reverse transcriptase. **c** Real-time PCR analysis of MPCs differentiated using protocol 1 and protocol 2. Differential gene expression analysis was performed for the main MPC genes (*PDX1*, *NKX6.1*, and *SOX9*). Fold-expression for each TF obtained for protocol 2 was normalized to that of protocol 1. **d** Flow cytometry analysis for the expression of NKX6.1 at the end of stage 4 of differentiation. All data shown are representative results from at least three independent experiments

The differentiation of hPSCs into pancreatic progenitors was performed using two different protocols. Differentiation was started when hPSCs reached 80% confluence. After removal of mTeSR1 media, the cells were washed with DPBS and then were treated with the differentiation media. We used two protocols as described in Fig. 1a. Protocol 1 was performed as previously described by Nostro et al. [10] with some modifications (Fig. 1a). These modifications were as follows: 1) MCDB 131 basal media (ThermoFisher Scientific, USA) was used for stages 1 and 2 instead of RPMI; 2) 2 μM CHIR99021 (Stemgent, USA) was used instead of Wnt3a for day 1 of differentiation; 3) 50 ng/ml NOGGIN (R&D Systems, USA) was used instead of dorsomorphin for stage 2; and 4) 0.25 μM SANT-1 (Sigma, USA) was used instead of KAAD-Cyclopamine for stage 3. Protocol 2 has recently been established in our laboratory [23]. In protocol 2, on day 4 of differentiation the cells were dissociated and replated at a density of 1.0–3.5 × 10^5^ cells/cm^2^ in the same media as in protocol 1 with the addition of 10 μM Y-27632 for 24 h and stage 3 was extended to 4 days (Fig. 1a) [23]. At the indicated time points cells were harvested and analyzed by reverse transcription polymerase chain reaction (RT-PCR) and immunostaining.

### RT-PCR and real-time PCR

The total RNA was isolated from the differentiated cells using the pure link™-RNA Minikit (ThermoFisher Scientific, USA) following the manufacturer’s instructions. The cDNA was synthesized using the superscript™ IV, First strand synthesis system kit (ThermoFisher Scientific). The genes were amplified by conventional PCR using PCR-Master mix (2×) (ThermoFisher Scientific) following the manufacturer’s protocol. The primer details are listed in Table 1. The RT-PCR products were analyzed by agarose gel electrophoresis. For quantitative RT-PCR (qRT-PCR), SYBR Green-based detection system (GoTaq qPCR Master Mix, Promega, USA) was used to quantify the expression level of mRNAs for *PDX1*, *NKX6.1*, and *SOX9*. Cycle threshold (CT) value was detected for each transcript and then normalized to the endogenous control *β-Actin*. Relative quantification was performed using the comparative ΔΔCT method for each transcript. The experiment was performed using the QuantStudio 7 Flex system (Applied Biosystems, CA, USA).

**Table 1:** The primer sequence list used for reverse transcription polymerase chain reaction analysis

Gene	Sequence	Product size
*GAPDH*	Forward: ACGACCACTTTGTCAAGCTCATTTC Reverse: GCAGTGAGGGTCTCTCTCTTCCTCT	132
*PDX1*	Forward: CGTCCAGCTGCCTTTCCCAT Reverse: CCGTGAGATGTACTTGTTGAATAGGA	178
*NKX6.1*	Forward: CTTCTGGCCCGGAGTGATG Reverse: GAAGAGAAAACACACGAGACCC	114
*NKX2.2*	Forward: CTTCTACGACAGCAGCGACAACCCG Reverse: CCTTGGAGAAAAGCACTCGCCGCTTT	221
*SOX9*	Forward: GACTACACCGACCACCAGAACTCC Reverse: GTCTGCGGGATGGAAGGGA	154
*PAX6*	Forward: CGAATTCTGCAGGTGTCCAA Reverse: ACAGACCCCCTCGGACAGTAAT	207
*HNF6*	Forward: GGACCTCAAGATAGCAGGTTTAT Reverse: CAGAATGCAGGTGAGCTAAGT	99
*GATA6*	Forward: AAGCGCGTGCCTTCATCA Reverse: TCATAGCAAGTGGTCTGGGC	157
*NGN3*	Forward: AGACGACGCGAAGCTCACC Reverse: AAGCCAGACTGCCTGGGCT	286
*FOXA2*	Forward: GGGAGCGGTGAAGATGGA Reverse: TCATGTTGCTCACGGAGGAGTA	89

### Immunofluorescence

Differentiated cells derived from hESCs and/or hiPSCs were washed two times with phosphate-buffered saline (PBS; ThermoFisher Scientific) and fixed in 4% paraformaldehyde in 0.1 M PBS (pH 7.4; Santa Cruz Biotechnology, USA) for 20 min. The cells were permeabilized for 15 min with 0.2% Triton X-100 (Sigma, USA) in PBS (PBST), and blocked for at least 2 h with 6% bovine serum albumin (BSA) in PBST at room temperature. The cells were then incubated at 4 °C overnight with the primary antibodies as described in Table 2. The cells were washed three times with tris-buffered saline with 0.3% Tween 20 (TBST) and then incubated with the following secondary antibodies: Alexa Fluor 488-labeled anti-guinea pig IgG, Alexa Fluor 568-labeled anti-mouse IgG, Alexa Fluor 488-labeled anti-rabbit IgG, or Alexa Fluor 568-labeled anti-rabbit IgG (1:500; ThermoFisher Scientific). Nuclei were counterstained with Hoechst 33,342 (1 μg/ml) (ThermoFisher Scientific). The plates were examined by Olympus IX53 inverted fluorescence microscopy (Japan).

**Table 2:** The details of the primary antibodies used for immunostaining

Antibody	Company	Catalog no.	Dilution
Rabbit anti-Chromogranin A	ThermoFisher Scientific	MA5-14536	1:4000
Rabbit anti-FOXA2	Cell Signaling Technology	3143	1:500
Rabbit anti-OCT4	Cell Signaling Technology	C30A3	1:500
Mouse anti-NKX6.1	DSHB	F55A12	1:2000
Mouse anti-NKX2.2	DSHB	74.5A5	1:2000
Guinea pig anti-PDX1	Abcam	Ab47308	1:1000
Mouse anti-SOX2	Cell Signaling Technology	L1D6A2	1:500
Mouse anti-SOX17	OriGene Technologies	CF500096	1:2000
Goat anti-NKX6.1	LifeSpan Biosciences	LS-C124275	1:2000
Goat anti-SOX9	R&D Systems	AF3075	1:500
Sheep anti-NGN3	R&D Systems	AF3444	1:1000
Mouse anti-cytokeratin 19	Merck Millipore	CBL198	1:500

### Flow cytometry

Cells at stage 4 of differentiation were fixed with 70% ethanol overnight. The fixed cells were blocked in 6% BSA in PBST for at least 2 h. The cells were incubated with mouse anti-NKX6.1 (1:100; DSHB) for 4 h at room temperature. The cells were incubated with Alexa-fluor 488 secondary antibody (1:200; ThermoFisher Scientific) for 40 min at room temperature. The analysis was performed using the BD Accuri C6 flow analyzer and the results were processed using FlowJo***.***

### Statistical analysis

All data shown are representative results from at least three independent experiments. Statistical significance was assessed by two-tailed Student’s *t* tests. Values of *P* < 0.05 were considered significant.

## Results

### Efficient differentiation of hPSCs into different populations of MPCs

Before starting the differentiation, the pluripotency of hPSCs was confirmed by examining the expression of SOX2 and OCT4 (Additional file 1: Figure S1A). To evaluate the formation of definitive endoderm (DE), we examined the expression of the specific markers for DE (SOX17 and FOXA2) using immunofluorescence at day 4 of differentiation. Furthermore, the pluripotency markers OCT4 and SOX2 were also examined to determine the differentiation efficiency. The differentiated cells showed relatively high expression of SOX17 and FOXA2 (Additional file 1: Figure S1B, C). On the other hand, the expression levels of OCT4 and SOX2 were dramatically reduced in the DE (Additional file 1: Figure S1B, C), indicating that the majority of cells had differentiated into DE and had lost their undifferentiated characteristics.

**Fig. 2:**
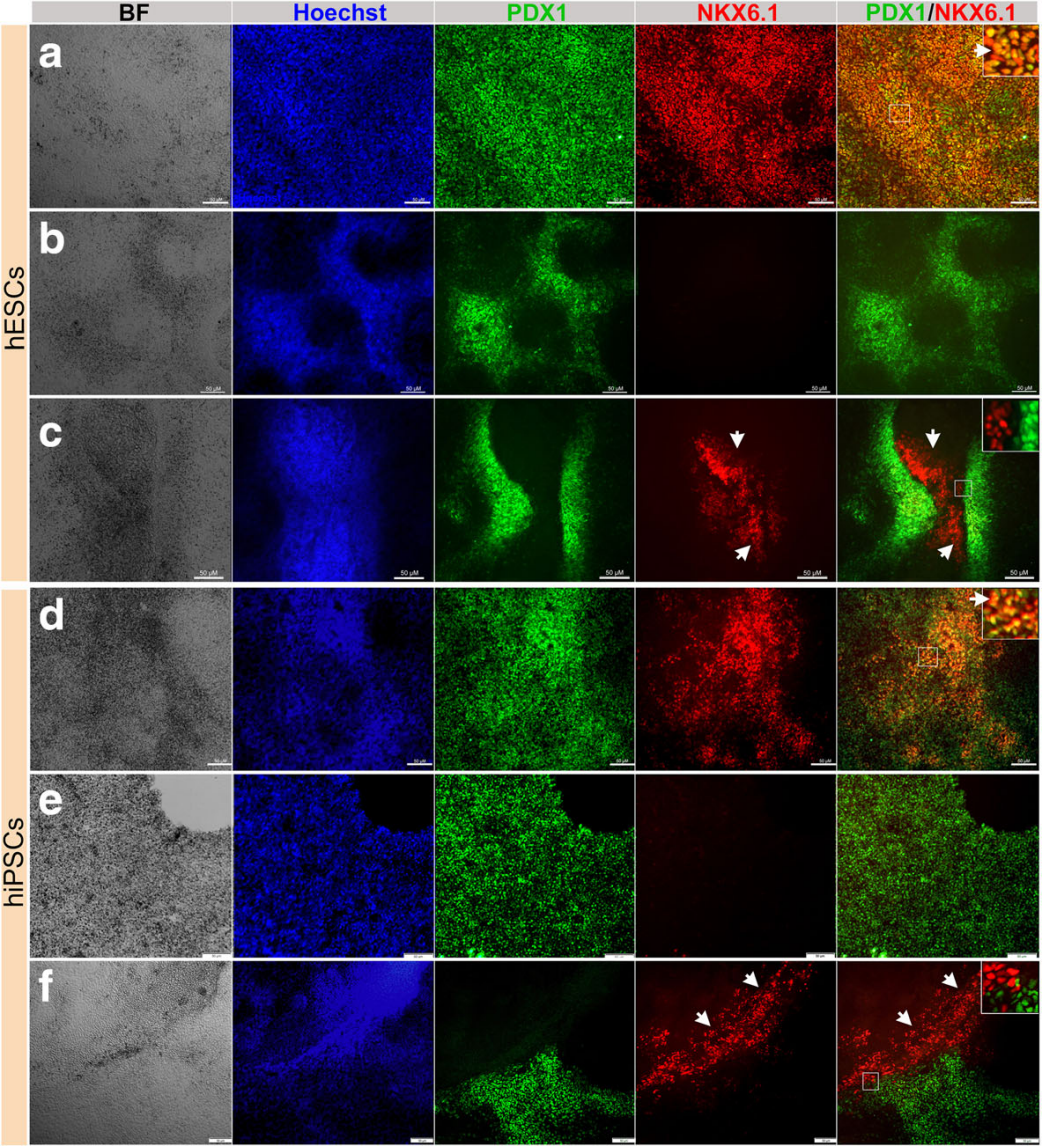
Differentiation of hPSC-derived definitive endoderm into different populations of MPCs using protocol 1. Immunofluorescence images of hPSC-derived MPCs showing expression of PDX1 (green) and NKX6.1 (red). MPCs generated from human embryonic stem cells (hESCs) (H1) (**a**–**c**) and human induced pluripotent stem cells (hiPSCs) (IMRI90) (**d**–**f**) showing three types of MPCs: **a**, **d** co-localization of PDX1 and NKX6.1 in MPCs (PDX1^+^/NKX6.1^+^), **b**, **e** expression of PDX1 in the absence of NKX6.1 (PDX1^+^/NKX6.1^−^), and **c**, **f** appearance of a novel NKX6.1 population lacking PDX1 expression (PDX1^−^/NKX6.1^+^). Squares indicate cells magnified in the insets to the right (**a**, **c**, **d**, **f**). Note clear distinction between the PDX1^−^/NKX6.1^+^ population and the other populations (PDX1^+^/NKX6.1^+^ and PDX1^+^/NKX6.1^−^). Arrowheads in (**a**, **d**) indicate PDX1^+^/NKX6.1^+^ cells and in (**c**, **f**) indicate PDX1^−^/NKX6.1^+^ cells. All data shown are representative results from at least three independent experiments. Scale bars = 50 μm

To further differentiate the DE into the pancreatic lineage, we applied two protocols as described in Methods (Fig. 1a). Following a monolayer-culture protocol (protocol 1) and a cell dissociation-based protocol (protocol 2), we successfully produced pancreatic progenitors with robust expression of PDX1^+^/NKX6.1^+^ cells, a vital characteristic that favors the differentiation of pancreatic progenitor cells into functional mature β cells (Fig. 1b–d, Fig. 2). The induction of pancreatic progenitors from hESC-H1 and hiPSC-IMR90 cell lines was confirmed by examining their gene expression profile with RT-PCR for stage-specific markers, including *PDX1*, *NKX6.1*, *SOX9*, *FOXA2*, *HNF6*, *NKX2.2*, *GATA6*, *NGN3*, and *PAX6* (Fig. 1b). Real-time PCR analysis for the main pancreatic progenitor markers showed a dramatic upregulation of *NKX6.1*, *PDX1*, and *SOX9* in the progenitors generated using protocol 2 [23] in comparison to protocol 1 (Fig. 1c) [10]. Similarly, flow cytometry analysis showed that the percentage of NKX6.1-positive cells was considerably higher in our protocol 2 (~86.5%) in comparison with protocol 1 from Nostro et al. (~64%) (Fig. 1d). These findings indicate the high efficiency of protocol 2. Furthermore, immunocytochemical analysis showed the presence of three distinct populations of pancreatic progenitors in terms of PDX1 and NKX6.1 expression (Fig. 2). The majority of the cells coexpressed the two TFs (PDX1^+^/NKX6.1^+^) (Fig. 2a, d). This PDX1^+^/NKX6.1^+^ population was evident in protocol 1 when stage 3 was shortened to 2 days (Fig. 2a, d). On the other hand, a subset of PDX1-expressing cells did not express NKX6.1 (PDX1^+^/NKX6.1^−^), which is a feature known for cells that favor the polyhormonal pancreatic lineage. This PDX1^+^/NKX6.1^−^ population was observed largely in MPCs generated using protocol 1 [10], when stage 3 duration was prolonged to 4 days (Fig. 2b, e). The expression levels of both TFs varied between the two cell lines. Interestingly, there was a subset of NKX6.1^+^ cells that, unusually, did not coexpress PDX1 (Fig. 2c, f). This third population (PDX1^−^/NKX6.1^+^) was noticed in MPCs generated from both cell lines. Following protocol 1, we noticed that this specific population of NKX6.1^+^ cells was often associated with the PDX1^+^ population. Sometimes it was sandwiched between PDX1^+^/NKX6.1^−^ populations (Fig. 2c) or the two populations were next to each other with clear segregations between both populations (Fig. 2f) in a monolayer arrangement of MPCs. Additionally, there were clear demarcations between PDX1^−^/NKX6.1^+^ and PDX1^+^/NKX6.1^−^ populations (Fig. 2). This population was noticed more in hiPSC-IMR90-derived MPCs compared to the H1-derived MPCs. These findings indicate that we could successfully generate a large number of PDX1^+^/NKX6.1^+^ MPCs. Moreover, we generated a novel population of PDX1^−^/NKX6.1^+^ cells that did not coexpress PDX1, a major marker for MPCs.

### Induction of cellular aggregations enhanced the PDX1^−^/NKX6.1^+^ population

**Fig. 3:**
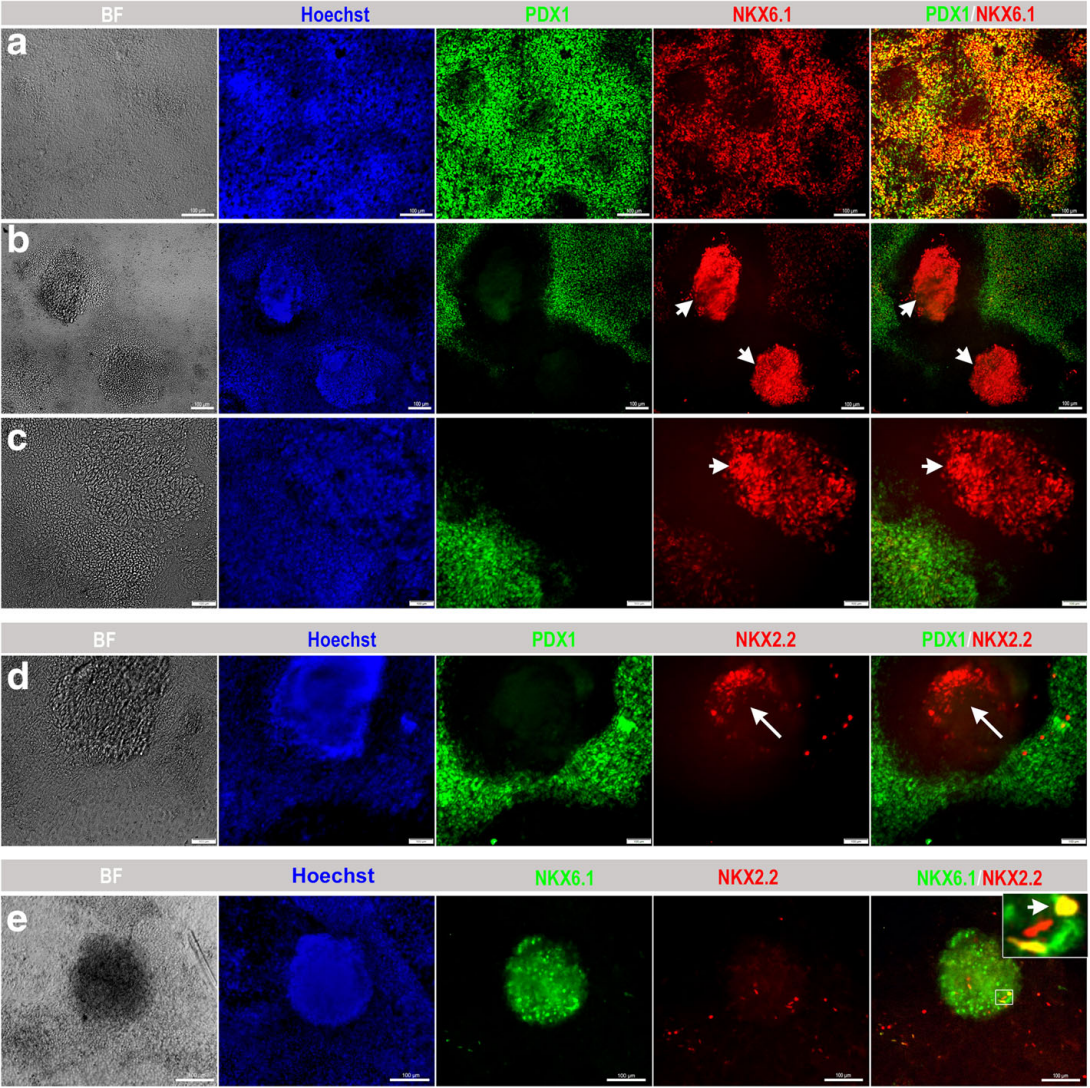
Differentiation of hPSCs into different populations of MPCs using protocol 2. Immunofluorescence analysis of MPCs generated using protocol 2 showing the generation of a large number of PDX1^+^/NKX6.1^+^ cells (**a**). Further investigation of the PDX1^−^/NKX6.1^+^ structures, which were generated after dissociation of the differentiated cells, showed that this population rearranged in 3D rounded structures (arrowheads) enclosed and surrounded by PDX1^+^/ NKX6.1^+^ expressing cells (**b**, **c**). These centrally located cellular 3D aggregates displayed an expression of NKX2.2 (arrow) in contrast to the surrounding cells that showed little or almost no NKX2.2 expression (**d**). NKX2.2 was expressed in some NKX6.1 expressing cells (arrowhead) in a 3D structure (**e**). The square indicates cells magnified in the insets on the right. All data shown are representative results from at least three independent experiments. Scale bars = 100 μm

It was previously reported that the formation of cellular aggregations could enhance the number of PDX1^+^/NKX6.1^+^ pancreatic progenitors [24]. Thus, we have recently established our own protocol based on cellular dissociation to obtain a large number of MPCs that can express both PDX1 and NKX6.1 [23]. The endodermal cells were dissociated on day 4 (beginning of stage 2) (Fig. 1a) during pancreatic progenitor differentiation and replated on new Matrigel-coated plates at a density of 1.0–3.5 × 10^5^ cells/cm^2^ [23]. Afterwards, we proceeded with the differentiation into pancreatic progenitors using the same media and components as used for protocol 1 [10] with the extension of stage 3 to 4 days instead of 2 days [23]. The replated cells formed cellular aggregates and, at the end of day 13 of differentiation, cells were evaluated for the expression of MPC stage-specific markers. Protocol 2 achieved a further increased expression of PDX1^+^/NKX6.1^+^ MPCs. Immunostaining showed a high number of cells coexpressing PDX1 and NKX6.1 (Fig. 3a). Using protocol 2, we were able to enrich the PDX1^−^/NKX6.1^+^ population; however, its distribution pattern was different. PDX1^−^/NKX6.1^+^ cells were notably distributed inside protruding three-dimensional (3D) aggregates that were surrounded by PDX1^+^/NKX6.1^+^ cells (Fig. 3b, c). Most of the 3D aggregates showed robust expression of NKX6.1 without expressing PDX1 (PDX1^−^/NKX6.1^+^) (Fig. 3b, c). However, moderate intensity of NKX6.1 expression was noticed in the surrounding population (PDX1^+^/NKX6.1^+^) (Fig. 3b, c). H1-hESCs showed a higher efficiency of differentiation than hiPSCs using protocol 2. Obtaining the 3D aggregates expressing PDX1^−^/NKX6.1^+^ was reproduced in our laboratory and the data presented here are a representative of more than ten independent differentiation experiments.

### The relationship between the PDX1^−^/NKX6.1^+^ population and other pancreatic markers

To understand and characterize the unique PDX1^−^/NKX6.1^+^ population, we examined the expression of NKX2.2 TF during pancreatic and endocrine development. In humans, it has been found that NKX2.2 is expressed in endocrine precursor cells, but is not expressed at earlier stages [7, 25]. Double immunostaining of PDX1 and NKX2.2 showed that a few 3D structures (obtained through protocol 2) that were constantly positive for NKX6.1 were also positive for NKX2.2 and negative for PDX1 as expected (Fig. 3d). We confirmed our findings by performing double-immunostaining for NKX6.1 and NKX2.2. As expected, the 3D structures were consistently positive for NKX6.1 and some cells within the structures were copositive for NKX2.2 (Fig. 3e). Since only a few 3D structures were positive for NKX2.2, it indicates that not all PDX1^−^/NKX6.1^+^ structures express NKX2.2 at this stage.

**Fig. 4:**
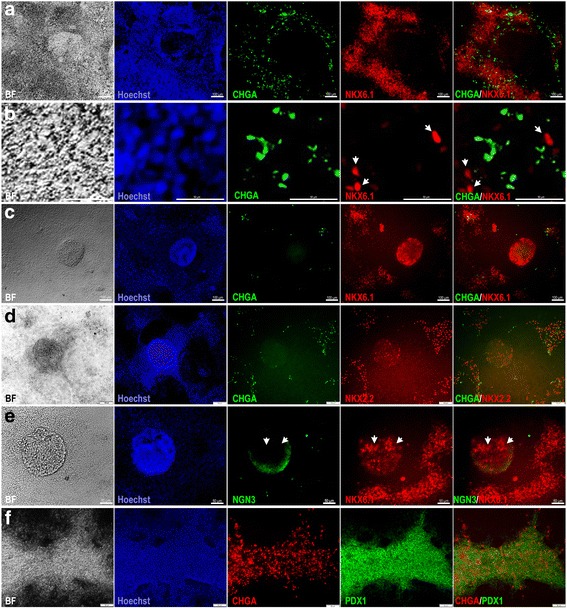
Characterization of endocrine markers in hPSC-derived MPCs. Immunofluorescence images of hPSC-derived MPCs using protocol 2 showing the expression of chromogranin A (CHGA; green) and NKX6.1 (red) in monolayer culture (**a**, **b**) and 3D aggregates (**c**). High magnification images showed that CHGA and NKX6.1 (arrowheads) were not colocalized in the same cells (**b**). The 3D aggregates showed no expression of CHGA, an early endocrine marker (**c**). **d** Immunofluorescence images of hPSC-derived MPCs showing expression of CHGA (green) and NKX2.2 (red). NKX2.2 was noticed in the 3D aggregates in the absence of CHGA, but both were expressed in the area surrounding the 3D aggregates. **e** Immunofluorescence images of hPSC-derived MPCs showing expression of neurogenin 3 (NGN3; green) and NKX6.1 (red). Note the absence of NGN3 in NKX6.1^+^ cells (arrowheads). **f** Immunofluorescence images of hPSC-derived MPCs showing expression of CHGA (red) and PDX1 (green). Note the presence of CHGA in the same areas expressing PDX1. All data shown are representative results from at least three independent experiments. Scale bars (**a**, **c**) = 100 μm and (**b**, **d**, **e**, **f**) = 50 μm

Next, we examined the expression of chromogranin A (CHGA), which is an early endocrine marker. Double immunostaining of CHGA and NKX6.1 showed that the areas that constantly express PDX1 and NKX6.1 were positive for CHGA (Fig. 4a, b). Although CHGA expression was detected in the same areas that expressed NKX6.1, we could not observe a clear colocalization of CHGA with NKX6.1 (Fig. 4b). In contrast, all 3D structures were negative for CHGA (Fig. 4c, d). Some CHGA-positive cells were noticed in the same areas that expressed NKX6.1 and NKX2.2 around the 3D structures (Fig. 4c, d). Furthermore, our results showed that the expression of NKX6.1 in the 3D structures was not colocalized with neurogenin 3 (NGN3) expression, the main marker for pancreatic endocrine progenitors (Fig. 4e). Double immunostaining of CHGA and PDX1 showed that CHGA was often expressed in the same areas that expressed PDX1 and some PDX1^+^ cells were positive for CHGA (Fig. 4f). These findings indicate that PDX1^−^/NKX6.1^+^ cells do not express the endocrine markers CHGA and NGN3.

**Fig. 5:**
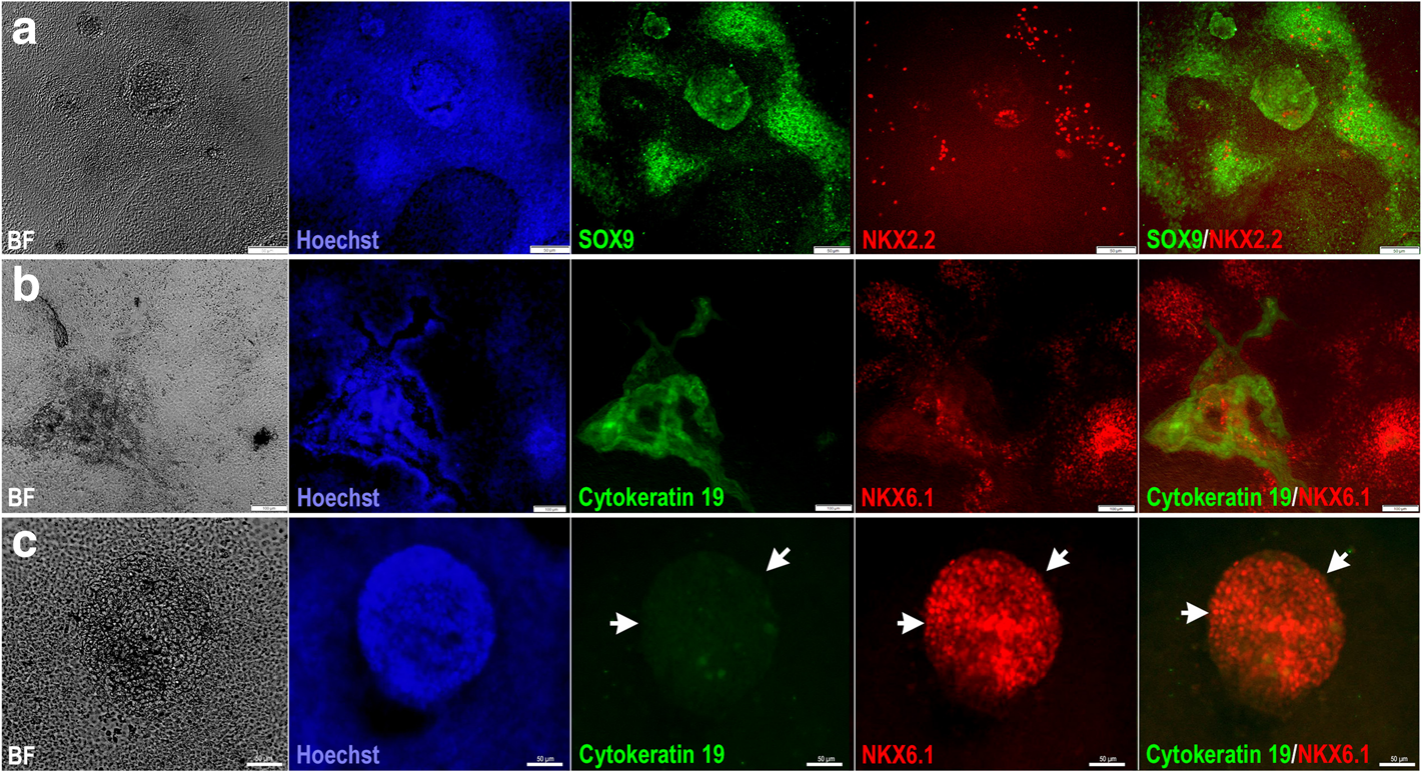
Characterization of ductal markers in hPSC-derived MPCs. **a** Immunofluorescence images of hPSC-derived MPCs showing the expression of SOX9 (green) and NKX2.2 (red) in 3D aggregates generated using protocol 2. Note the expression of SOX9 in the 3D aggregates. **b**, **c** Immunofluorescence images of hPSC-derived MPCs showing expression of cytokeratin 19 (green) and NKX6.1 (red). The NKX6.1 expression was noticed in the cells adjacent to cytokeratin-positive cells in a monolayer arrangement of pancreatic progenitors (**b**); however, cytokeratin 19 was completely absent in the 3D aggregates which were positive for NKX6.1 (arrowheads) (**c**). All data shown are representative results from at least three independent experiments. Scale bars (**a**, **c**) = 100 μm and (**b**) = 50 μm

Next, we investigated whether the PDX1^−^/NKX6.1^+^ population expressed ductal markers. Our results demonstrated that SOX9 was expressed in the 3D structures (protocol 2) as well as the area surrounding this population (Fig. 5a). Since SOX9 is not exclusive for pancreatic duct cells, we also examined cytokeratin 19, which is a specific marker for differentiated ductal cells (Fig. 5b, c). For pancreatic progenitors (protocol 2) arranged in monolayer sheets, NKX6.1 expression was noted in the vicinity of cytokeratin 19-positive cells but was not colocalized in the same cells (Fig. 5b). However, the 3D structures (protocol 2) that were positive for NKX6.1 were completely negative for cytokeratin 19 (Fig. 5c). These results indicate that the PDX1^−^/NKX6.1^+^ population is not involved in the development of pancreatic ductal epithelium.

The specificity of the antibodies used in this study has previously been confirmed in several studies [13, 26, 27]. In addition, we have used control experiments to confirm the specificity.

## Discussion

The eminent shortage of pancreatic islet donors is a significant obstacle for islet transplantation therapy and for studying human pancreatic development. hPSC technology is considered a promising approach that can be used to provide an unlimited supply of human pancreatic cells. However, generation of a large number of fully functional pancreatic β cells responding to glucose in vitro still requires further studies. To improve the differentiation efficiency, it is crucial to study the molecular characteristics specifying each type of pancreatic lineage during human pancreatic development. In an attempt to understand the pancreatic endocrine specification in humans, we differentiated hPSCs into different populations of MPCs expressing specific TFs. We found that there are two distinct populations of NKX6.1^+^ cells that can be generated in hPSC-derived MPCs in vitro. One of these NKX6.1 populations coexpressed PDX1 (PDX1^+^/NKX6.1^+^) which has also been generated by other groups [10, 18, 19] and is known to be the precursor of the functional insulin-secreting cells [28, 29]. The other novel NKX6.1 population which has recently been discovered by our team [23] did not express PDX1 (PDX1^−^/NKX6.1^+^). Interestingly, we noticed that the PDX1^−^/NKX6.1^+^ population was uniquely surrounded by PDX1^+^/NKX6.1^+^ cells. These observations may suggest that the existence of two different populations of NKX6.1^+^ MPCs during human pancreas development, in contrast to the single previously reported population (PDX1^+^/NKX6.1^+^). The fate of this novel population is still unknown.

The relationship between NKX6.1 and the endocrine progenitor marker NGN3 in the MPC stage during pancreatic differentiation determines the endocrine cell fate. It is known that PDX1^+^ MPCs can generate all types of pancreatic lineages [30] and NKX6.1 induction in those progenitors (PDX1^+^/NKX6.1^+^) directs them into endocrine and/or ductal cells only [31]. It has been reported that the expression of NGN3 at the early MPC stage in the absence of NKX6.1 leads to their differentiation into polyhormonal endocrine cells (insulin^+^/NKX6.1^−^). In contrast, the existence of NKX6.1 before starting the expression of NGN3 leads to their differentiation into monohormonal β cells (insulin^+^/NKX6.1^+^) [13]. Interestingly, transplantation of hPSC-derived MPCs containing a low number of NKX6.1^+^ cells (25%) and a high number of cells expressing endocrine progenitor markers (60%) have differentiated into α cells and immature β cells in vivo [14]. However, MPCs containing a high number of NKX6.1 (80%) and only 11% endocrine progenitor cells differentiated mainly into functional glucose-responsive β cells [14]. In agreement with these findings, our data showed the absence of NGN3 and CHGA expression (endocrine markers) in the PDX1^−^/NKX6.1^+^ population, indicating that the NKX6.1 expression is inversely proportional to the expression of the early endocrine markers at the MPC stage. Moreover, the absence of the early endocrine marker in the NKX6.1^+^ structures in the MPCs may allow those progenitors to differentiate into functional pancreatic β cells.

Furthermore, our results showed that some 3D structures that are consistently positive for NKX6.1 (PDX1^−^/NKX6.1^+^) expressed NKX2.2 in the absence of PDX1 expression. This further provides evidence that at least some of this uncharacterized population may be mature endocrine progenitors. NKX2.2 has been shown to act downstream of the key endocrine regulator NGN3, for which the structures stained negative [32]. In contrast to NKX6.1 expression, NKX2.2 is not expressed at early stages of pancreatic progenitors but rather during the late endocrine stage in humans [7, 25]. It has been reported that NKX2.2 is required for all four endocrine cell types in human fetal endocrine cells and its expression level defines α- and β-cell identities [22, 33, 34]; however, NKX6.1 is exclusive and specific to β cells [21, 22]. The fact that no PDX1 expression was noticed in the PDX1^−^/NKX6.1^+^ population is comparable with the temporary loss of PDX1 expression noticed during human pancreatic endocrine cell maturation as it has been revealed that PDX1 is re-expressed in the mature β cells [22]. This strongly suggests that at least some of the unique PDX1^−^/NKX6.1^+^ population may be precursors for β cells and are not specified towards the other endocrine cell lineages.

Despite the combination of some TFs coexpressed by our uniquely generated MPC population defining them as β-cell precursors, the absence of PDX1 allows debate on the origin of this progenitor population and its fate. During embryogenesis, NKX6.1^+^ cells can generate pancreatic ductal epithelium and endocrine precursors [25]. Previous studies reported that pancreatic endocrine cells often appear adjacent to duct-like structures during pancreatic development [35–37], and the endocrine islet cells may originate from the pancreatic duct at a certain stage [38]. This suggests that it is possible that our PDX1^−^/NKX6.1^+^ cells are a subpopulation of ductal epithelium, supported by the expression of SOX9 in the 3D structures. However, our data showed that cytokeratin 19, a ductal marker, was not coexpressed with NKX6.1 in the 3D structures. These findings indicate that the PDX1^−^/NKX6.1^+^ population obtained in this study may not be involved in the development of pancreatic ductal epithelium.

The data presented here indicate that the role of NKX6.1 during human pancreatic development is still unclear and that further studies are needed. Discovery of a novel MPC population suggests that not all data obtained from animal models can be applied to humans since there are several differences in TFs and genes regulating pancreatic development between humans and animals [39–42]. Studies on the human embryo at early stages of development are rare due to the shortage of pancreatic samples and ethical concerns; thus, most of the available data have been generated from samples taken after 7–8 weeks during embryogenesis, suggesting the stages after MPCs have already been developed [22, 43–47]. Therefore, our work supports the hypothesis that hPSCs may be a possible alternative approach to study early human pancreatic development since they allow research directly on human cells without the ethical concerns. Therefore, our future studies will continue to completely characterize this novel population and identify its fate and role in pancreatic development. Purification and characterization of each subpopulation of NKX6.1^+^ MPCs through identifying surface markers coupled with genomic assays would help in identifying the regulatory network associated with each type of NKX6.1 population.

## Conclusions

To conclude, we have shown here for the first time that hPSCs can be differentiated into two distinct MPC populations of NKX6.1^+^ cells that may have the potential to generate endocrine islet cells. Strikingly, in this study we noticed that all PDX1^−^/NKX6.1^+^ cells were surrounded, in between, or adjacent to other MPC populations, indicating that these two populations may normally develop close to each other during pancreas development in humans. While the paradigm still classifies PDX1^+^/NKX6.1^+^ cells as the main progenitor population that can differentiate into monohormonal insulin-secreting cells [10, 18, 19, 28, 29], it is noteworthy to gauge the developmental potential of this novel PDX1^−^/NKX6.1^+^ MPC population to generate insulin-secreting cells in order to determine their clinical relevance and application for transplantation therapy. Our findings presented here may contribute to improve our understanding of human pancreas development and open new avenues toward identifying new roles for NKX6.1 during pancreatic development.

## Additional file

Additional file 1: **Figure S1.** Expression of endodermal markers in hPSC-derived definitive endoderm (DE). (A) Immunofluorescence images showing the expression of SOX2 (red) and OCT4 (green) in hESCs before starting the differentiation process. Representative images of hESC-derived DE expressing high levels of SOX17 (B) and FOXA2 (C) at stage 1 of differentiation. Note the dramatic reduction in the expression of the main pluripotency markers (OCT4 and SOX2) after differentiation. Nuclei are labeled with Hoechst. All data shown are representative results from at least three independent experiments. Scale bars = 50 μm. (JPEG 1645 kb)

## Abbreviations

3D: Three-dimensional
BSA: Bovine serum albumin
CHGA: Chromogranin A
DE: Definitive endoderm
hESC: human embryonic stem cell
hiPSC: Human induced pluripotent stem cell
hPSC: Human pluripotent stem cell
MPC: Multipotent progenitor cell
NGN3: Neurogenin 3
NKX6.1: NK6 homeobox transcription factor related, locus 1
PBS: Phosphate-buffered saline
PDX1: Pancreatic and duodenal homeobox 1
RT-PCR: Reverse transcription polymerase chain reaction
T1D: Type 1 diabetes
T2D: Type 2 diabetes
TF: Transcription factor

## References

[1] Ahren B (2005). Type 2 diabetes, insulin secretion and beta-cell mass. Curr Mol Med..

[2] Leibowitz G, Kaiser N, Cerasi E (2011). Beta-cell failure in type 2 diabetes. J Diabetes Investig..

[3] Shapiro AM (2011). State of the art of clinical islet transplantation and novel protocols of immunosuppression. Curr Diab Rep..

[4] Tyden G, Reinholt FP, Sundkvist G (1996). Recurrence of autoimmune diabetes mellitus in recipients of cadaveric pancreatic grafts. N Engl J Med..

[5] Abdelalim EM, Bonnefond A, Bennaceur-Griscelli A (2014). Pluripotent stem cells as a potential tool for disease modelling and cell therapy in diabetes. Stem Cell Rev..

[6] Abdelalim EM, Emara MM (2015). Advances and challenges in the differentiation of pluripotent stem cells into pancreatic beta cells. World J Stem Cells..

[7] Oliver-Krasinski JM, Stoffers DA (2008). On the origin of the beta cell. Genes Dev..

[8] Van Hoof D, D'Amour KA, German MS (2009). Derivation of insulin-producing cells from human embryonic stem cells. Stem Cell Res..

[9] Mfopou JK, Chen B, Mateizel I (2010). Noggin, retinoids, and fibroblast growth factor regulate hepatic or pancreatic fate of human embryonic stem cells. Gastroenterology.

[10] Nostro MC, Sarangi F, Yang C (2015). Efficient generation of NKX6-1+ pancreatic progenitors from multiple human pluripotent stem cell lines. Stem Cell Reports..

[11] Kroon E, Martinson LA, Kadoya K (2008). Pancreatic endoderm derived from human embryonic stem cells generates glucose-responsive insulin-secreting cells in vivo. Nat Biotechnol..

[12] Bruin JE, Rezania A, Xu J (2013). Maturation and function of human embryonic stem cell-derived pancreatic progenitors in macroencapsulation devices following transplant into mice. Diabetologia..

[13] Russ HA, Parent AV, Ringler JJ (2015). Controlled induction of human pancreatic progenitors produces functional beta-like cells in vitro. EMBO J..

[14] Rezania A, Riedel MJ, Wideman RD (2011). Production of functional glucagon-secreting alpha-cells from human embryonic stem cells. Diabetes..

[15] Rezania A, Bruin JE, Xu J (2013). Enrichment of human embryonic stem cell-derived NKX6.1-expressing pancreatic progenitor cells accelerates the maturation of insulin-secreting cells in vivo. Stem Cells.

[16] Polidori GP. Macroencapsulation of pancreatic progenitors: a new era in diabetes therapy? J Clin Dev Biol. 2016;1:1.

[17] Tuch BE, Hughes TC, Evans MD (2011). Encapsulated pancreatic progenitors derived from human embryonic stem cells as a therapy for insulin-dependent diabetes. Diabetes Metab Res Rev..

[18] Pagliuca FW, Millman JR, Gurtler M (2014). Generation of functional human pancreatic beta cells in vitro. Cell..

[19] Rezania A, Bruin JE, Arora P (2014). Reversal of diabetes with insulin-producing cells derived in vitro from human pluripotent stem cells. Nat Biotechnol..

[20] Al-Khawaga S, Memon B, Butler AE, et al. Pathways governing development of stem cell-derived pancreatic beta cells: lessons from embryogenesis. Biol Rev Camb Philos Soc. 2018;93:364–89. 10.1111/brv.12349 28643455

[21] Schisler JC, Jensen PB, Taylor DG (2005). The Nkx6.1 homeodomain transcription factor suppresses glucagon expression and regulates glucose-stimulated insulin secretion in islet beta cells. Proc Natl Acad Sci U S A.

[22] Lyttle BM, Li J, Krishnamurthy M (2008). Transcription factor expression in the developing human fetal endocrine pancreas. Diabetologia..

[23] Memon B, Karam M, Al-Khawaga S (2018). Enhanced differentiation of human pluripotent stem cells into pancreatic progenitors co-expressing PDX1 and NKX6.1. Stem Cell Res Ther.

[24] Toyoda T, Mae S, Tanaka H (2015). Cell aggregation optimizes the differentiation of human ESCs and iPSCs into pancreatic bud-like progenitor cells. Stem Cell Res..

[25] Jennings RE, Berry AA, Kirkwood-Wilson R (2013). Development of the human pancreas from foregut to endocrine commitment. Diabetes..

[26] Kelly OG, Chan MY, Martinson LA (2011). Cell-surface markers for the isolation of pancreatic cell types derived from human embryonic stem cells. Nat Biotechnol..

[27] Takeuchi H, Nakatsuji N, Suemori H (2014). Endodermal differentiation of human pluripotent stem cells to insulin-producing cells in 3D culture. Sci Rep..

[28] Nelson SB, Schaffer AE, Sander M (2007). The transcription factors Nkx6.1 and Nkx6.2 possess equivalent activities in promoting beta-cell fate specification in Pdx1+ pancreatic progenitor cells. Development..

[29] Sander M, Sussel L, Conners J (2000). Homeobox gene Nkx6.1 lies downstream of Nkx2.2 in the major pathway of beta-cell formation in the pancreas. Development..

[30] Gu G, Brown JR, Melton DA (2003). Direct lineage tracing reveals the ontogeny of pancreatic cell fates during mouse embryogenesis. Mech Dev..

[31] Schaffer AE, Freude KK, Nelson SB (2010). Nkx6 transcription factors and Ptf1a function as antagonistic lineage determinants in multipotent pancreatic progenitors. Dev Cell..

[32] Churchill AJ, Gutierrez GD, Singer RA, et al. Genetic evidence that Nkx2.2 acts primarily downstream of Neurog3 in pancreatic endocrine lineage development. Elife. 2017;6. 10.7554/eLife.20010 PMC5224921 28071588

[33] Doyle MJ, Loomis ZL, Sussel L (2007). Nkx2.2-repressor activity is sufficient to specify alpha-cells and a small number of beta-cells in the pancreatic islet. Development..

[34] Jorgensen MC, Ahnfelt-Ronne J, Hald J (2007). An illustrated review of early pancreas development in the mouse. Endocr Rev..

[35] Dudek RW, Lawrence IE, Hill RS (1991). Induction of islet cytodifferentiation by fetal mesenchyme in adult pancreatic ductal epithelium. Diabetes..

[36] Pang K, Mukonoweshuro C, Wong GG (1994). Beta cells arise from glucose transporter type 2 (Glut2)-expressing epithelial cells of the developing rat pancreas. Proc Natl Acad Sci U S A..

[37] Teitelman G, Alpert S, Polak JM (1993). Precursor cells of mouse endocrine pancreas coexpress insulin, glucagon and the neuronal proteins tyrosine hydroxylase and neuropeptide Y, but not pancreatic polypeptide. Development..

[38] Bonner-Weir S, Taneja M, Weir GC (2000). In vitro cultivation of human islets from expanded ductal tissue. Proc Natl Acad Sci U S A..

[39] De Vos A, Heimberg H, Quartier E (1995). Human and rat beta cells differ in glucose transporter but not in glucokinase gene expression. J Clin Invest..

[40] Hay CW, Docherty K (2006). Comparative analysis of insulin gene promoters: implications for diabetes research. Diabetes..

[41] McDonald TJ, Tu E, Brenner S (1994). Canine, human, and rat plasma insulin responses to galanin administration: species response differences. Am J Phys..

[42] Steiner DJ, Kim A, Miller K (2010). Pancreatic islet plasticity: interspecies comparison of islet architecture and composition. Islets..

[43] Castaing M, Duvillie B, Quemeneur E (2005). Ex vivo analysis of acinar and endocrine cell development in the human embryonic pancreas. Dev Dyn..

[44] Piper Hanley K, Hearn T, Berry A (2010). In vitro expression of NGN3 identifies RAB3B as the predominant Ras-associated GTP-binding protein 3 family member in human islets. J Endocrinol..

[45] Polak M, Bouchareb-Banaei L, Scharfmann R (2000). Early pattern of differentiation in the human pancreas. Diabetes..

[46] Piper K, Brickwood S, Turnpenny LW (2004). Beta cell differentiation during early human pancreas development. J Endocrinol..

[47] Sarkar SA, Kobberup S, Wong R (2008). Global gene expression profiling and histochemical analysis of the developing human fetal pancreas. Diabetologia..

