# Supplementary figures and images for: Differentiation of human pluripotent stem cells into two distinct NKX6.1 populations of pancreatic progenitors
Source: Stem Cell Res Ther. 2018 Apr 3;9:83. doi: 10.1186/s13287-018-0834-0 (PMC5883581; doi:10.1186/s13287-018-0834-0)

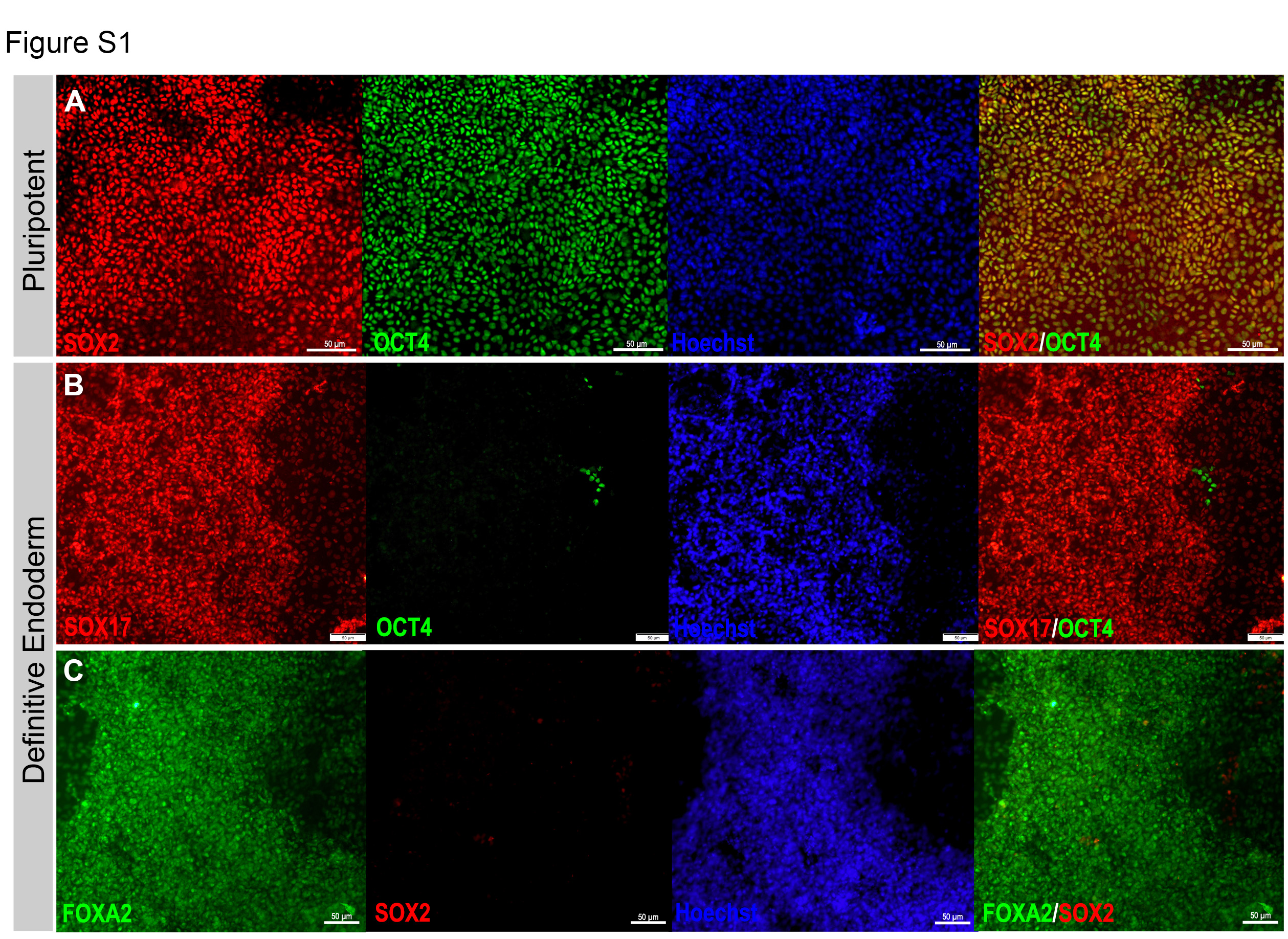

Supplement: Supplementary file 1 — Figure S1. Expression of endodermal markers in hPSC-derived definitive endoderm (DE). (A) Immunofluorescence images showing the expression of SOX2 (red) and OCT4 (green) in hESCs before starting the differentiation process. Representative images of hESC-derived DE expressing high levels of SOX17 (B) and FOXA2 (C) at stage 1 of differentiation. Note the dramatic reduction in the expression of the main pluripotency markers (OCT4 and SOX2) after differentiation. Nuclei are labeled with Hoechst. All data shown are representative results from at least three independent experiments. Scale bars = 50 μm. (JPEG 1645 kb) [file 13287_2018_834_MOESM1_ESM.jpg]
